# The nature and nurture of resilience—reactions of trizygotic triplet minors to their father’s death

**DOI:** 10.1007/s40211-022-00434-z

**Published:** 2022-10-27

**Authors:** Doris Mayerhofer, Gertrude Bogyi, Christine Koska, Regina Rüsch, Judith Thaller, Katrin Skala

**Affiliations:** 1 Department of Child- and Adolescent Psychiatry, Medical University of Vienna, Spitalgasse 23, 1090 Vienna, Austria; 2 Ambulatorium “Die Boje” (“The Buoy”), Vienna, Austria

**Keywords:** Grief, Loss, Intellectual disability, Psychological adaptation, Environmental factors, Trauer, Verlust, Intellektuelle Behinderung, Psychologische Adaptation, Umgebungsfaktoren

## Abstract

**Background:**

Parental bereavement during childhood is associated with an elevated risk for the development of psychiatric problems. This paper seeks to provide insight into the adjustment process of trizygotic triplets dealing with their father’s death, thus, trying to give an impression of the individual nature of grief and resilience.

**Methods:**

We examined 11-year-old trizygotic triplets (2 boys and 1 girl) concerning behavioral problems (CBCL/6-18R, YSR/11-18R), posttraumatic stress disorder (UCLA PTSD Index for DSM‑5, UPID), depression (“Depressionsinventar für Kinder und Jugendliche,” DIKJ), and fear (“Phobiefragebogen für Kinder und Jugendliche,” PHOKI) shortly after their father’s death and 6 months later. The girl was developmentally delayed and had moderate intellectual disability, while her brothers’ development was age-appropriate.

**Results:**

The triplets showed very different adjustment to the traumatic event. While the boys showed less psychopathological response to their loss shortly after their father’s death and adjusted physiologically during the following 6 months, their sister scored high in almost all dimensions and still presented with notable psychopathological symptoms at the 6‑month follow-up.

**Conclusion:**

Outcomes differ distinctly despite objectively equal risk and protective factors. While it is known that above average intellectual abilities represent a protective factor for posttraumatic stress, these results show that intellectual retardation might be a prominent yet understudied risk factor in dealing with postbereavement psychopathology in children.

**Supplementary Information:**

The online version of this article (10.1007/s40211-022-00434-z) contains supplementary material, which is available to authorized users.

## Abstract (de)

**Hintergrund:**

Der Verlust eines Elternteils in der Kindheit ist mit einem erhöhten Risiko für die Entwicklung psychiatrischer Probleme verbunden. Die vorliegende Arbeit soll einen Einblick in den Anpassungsprozess von trizygotischen Drillingen geben, die den Tod ihres Vaters zu bewältigen haben, und so einen Eindruck der Individualität von Trauer und Resilienz vermitteln.

**Methoden:**

Wir untersuchten 11-jährige trizygotische Drillinge (2 Jungen und 1 Mädchen) hinsichtlich Verhaltensauffälligkeiten (CBCL/6-18R, YSR/11-18R), posttraumatischer Belastungsstörung (UCLA PTSD Index for DSM‑5, UPID), Depression („Depressionsinventar für Kinder und Jugendliche“, DIKJ) und Angst („Phobiefragebogen für Kinder und Jugendliche“, PHOKI) kurz nach dem Tod des Vaters und 6 Monate danach. Das Mädchen hatte eine Entwicklungsverzögerung und eine leichte Intelligenzminderung, der Entwicklungsstand ihrer Brüder dagegen war altersentsprechend.

**Ergebnisse:**

Die Drillinge zeigten eine sehr unterschiedliche Verarbeitung des traumatischen Ereignisses. Während die Jungen kurz nach dem Tod ihres Vaters eine geringere psychopathologische Reaktion auf den Verlust und in den folgenden 6 Monaten eine physiologische Anpassung zeigten, erzielte ihre Schwester in fast allen Dimensionen hohe Werte und wies bei der Follow-up-Untersuchung nach 6 Monaten immer noch auffällige psychopathologische Symptome auf.

**Schlussfolgerung:**

Trotz der objektiv gleichen Risiko- und Schutzfaktoren unterscheiden sich die Folgen deutlich. Während überdurchschnittliche intellektuelle Fähigkeiten als protektiver Faktor für posttraumatischen Stress erwiesen sind, zeigen die vorliegenden Ergebnisse, dass intellektuelle Retardierung ein wichtiger, aber bisher nicht adäquat erforschter Risikofaktor im Kontext der kindlichen posttraumatischen Psychopathologie sein könnte.

## Background research

### Evidence before the study

We searched PubMed Central for all studies or reports presenting data on grief in children with intellectual disability, using the terms “grief” and “children” and “intellectual disability” from database inception to March 20, 2021. No language restrictions were applied. Our search returned 58 publications. Of these 54 papers highlighted the grief of parents of disabled children, only 2 papers (published in 1978 and 1986) dealt with intellectually disabled children as mourners. Although research on grief in intellectually impaired children is obviously scarce, it is well known that, in addition to female gender and extraversion, above average intellectual abilities represent a core characteristic of resilience.

### Added value of this study

This study reports data from trizygotic triplets, one of which has an intellectual disability, who are facing their father’s death. The great differences in their individual mourning processes and, above all, the structurally different course of the symptoms of the girl with an intellectual disability compared to their intellectually average-endowed brothers support the theory that intellectual disability is a risk factor for the development of pathological grief reactions.

### Implications of all the available evidence

This report clearly emphasizes the individual nature of grief and resilience. It shows that developmental disability and associated difficulties regarding the understanding of the event or the impaired capacity to cope seems to be a major risk factor in the adaptation to the loss of a loved one. An impaired ability to understand death as well as missing emotional skills to express distress may lead to complicated grieving processes in children and adolescents. These findings might contribute to an increase of awareness and understanding of grieving children with intellectual disability. Further research is however needed to promote understanding of the grieving processes in this population, with the objective of generating evidence-based guidelines concerning grief education and psychological care for subjects with developmental delay or intellectual deficiency.

## Introduction

Adverse childhood events are known risk factors for the exhibition of serious long-term consequences in both mental [1, 2] and physical health [3, 4] with a sizeable impact on the burden of disease [2]. Like other adverse events, parental bereavement during childhood is, regardless of the cause of death [5], associated with a higher risk of psychiatric problems such as posttraumatic stress disorder (PTSD), depression, anxiety, substance abuse or suicidal behavior [6, 7]. Most children, however, do not develop complicated grief reactions [8]. Factors such as a child’s emotional development, the cognitive ability to understand the event, the circumstances of death and the relationship to the deceased parent are known to influence the aptitude to adjust to the loss of an attachment figure [9]. It is known that, in the context of parental loss, the surviving parent has a crucial role for the child’s further development [5, 10], but the controversy on nature and nurture of resilience in this context is still unresolved.

Decades ago, findings of “competent” or “invincible” children who successfully adapt to different adversities led to the first description of what has hence become known by the term *resilience* [11]. While good intellectual capacity is a recognized protective factor [11], there are indications that intellectual disability (ID) is a risk factor for the development of traumatic grief reactions or other psychiatric symptoms [12, 13]. However, the situation of grieving children with ID has not yet been examined in a sustainable manner.

The current paper seeks to provide insight into the different adjustment processes of trizygotic triplets dealing with their father’s death, thus, trying to give an impression of the individual nature of grief and resilience.

## Methods

### Design, study site and subjects

Aiming to assess factors of resilience in minors with a history of loss, violence or divorce, we conducted a prospective study on children experiencing the death of an attachment figure. The study site was a low threshold outpatient clinic (“The Buoy”, www.die-boje.at) located in Vienna, Austria. This clinic offers short-term psychotherapy to children after the experience of traumatic events. Treatment expenses are covered by the mandatory health insurance so access is free to everyone. Investigation was performed recently after the children’s first appointment (V1) and at a 6-month follow-up (V2).

The data presented here derive from 11-year-old trizygotic triplets who presented at the clinic shortly after their father had died from cancer.

### Instruments

At both appointments, we conducted a thorough investigation of the children’s mental situation examining them for behavioral problems (CBCL/6-18R and YSR/11-18R), posttraumatic stress disorder (UCLA PTSD Index for DSM‑5, UPID), depression (“Depressionsinventar für Kinder und Jugendliche,” DIKJ) and fears (“Phobiefragebogen für Kinder und Jugendliche,” PHOKI). We also carried out a qualitative Interview on the children’s relationship with their most important attachment figures. For details on the instruments used, see Suppl. 1.

## Results

### Triplet A

Triplet A is a girl with moderate intellectual disability and developmental delay who appears very maidenly. Initially, she seems to be shy but throughout the first appointment she relaxed and opened.

**Fig. 1:**
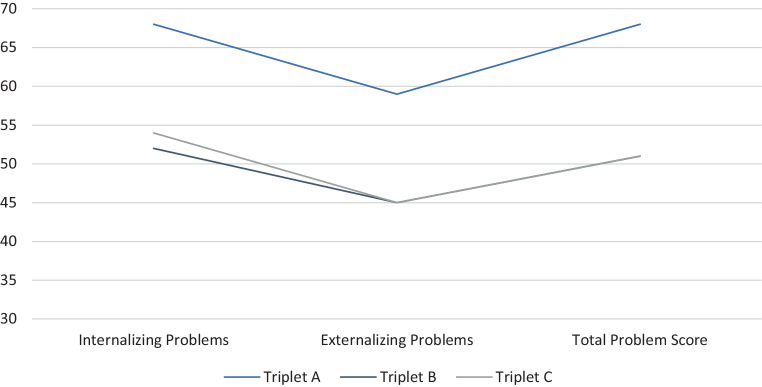
Results of YSR 11/18 at V1

**Fig. 2:**
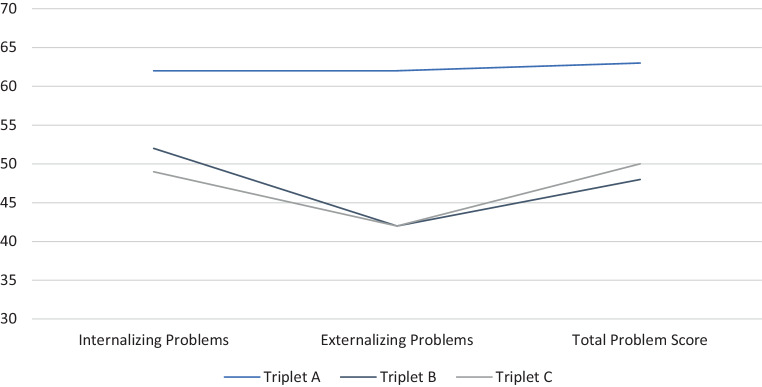
Results of YSR 11/18 at V2

The broadband scores of self-reported behavioral problems (YSR/11-18R) decreased throughout the 6‑month period (Figs. 1 and 2). At V1, she exhibited clinically relevant behavioral problems at the narrow band scale “Anxious and Depressed” which resulted in an elevated “Internalizing Problems” score, further the elevated narrowband score “Aggressive Behavior” contributed to clinically relevant “Externalizing Problems”. Additionally, pronounced “Social Problems” and “Thought Problems” accounted for the clinically relevant “Total Problem Score”. Six months later, she still exhibited clinically relevant suffering on the scores “Anxious and Depressed”, “Social Problems” and “Thought Problems”, again resulting in an elevated “Total Problem Score”.

**Fig. 3:**
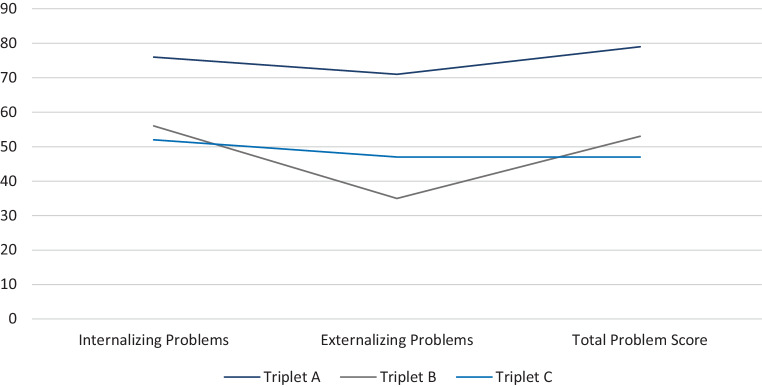
Results of CBCL at V2

The mother described yearning, sadness and separation anxiety as well as clinically relevant behavioral problems at the broad band scores of both internalizing and externalizing problems (CBCL/6-18R). On the level of the narrow-band scores, all scores revealed clinically relevant behavioral problems with “Somatic Problems” and “Aggressive Behavior” being only marginally elevated. The girl had also reported suicidal ideation (Fig. 3).

During the observation period DIKJ, T‑value decreased from T = 63, equaling borderline depressive symptoms, to T = 54, implying a reduction of depressive symptoms beyond clinical relevance. Changes in PHOKI scores reveal distinct improvement concerning anxiety. At V1 Total Score was Stanine = 9 with all subscale scores indicating significant suffering from fear. At V2, PHOKI total score had decreased to Stanine = 6, which is considered average. At the subscales “Fear of Menace”, “Fear of Animals” and “Fear of Medical Interventions”, levels however remain marginally elevated (Stanine = 7).

UPID indicates relevant suffering from a traumatic event. Concerning this matter, triplet A refers to the death of her father and reports clinically relevant suffering from symptoms of hyperarousal, avoidance and re-experiencing the traumatic situation. She described compulsive and recurrent intrusive thoughts about her father’s buried body. Total score of trauma-related symptomatology is 28, considered clinically relevant.

When asked about grown-up attachment figures, triplet A spontaneously refers to her mother and her father. She describes the relationship to her mother as stable, caring and loving. Further, she states that her mother and her brothers care for her very well. At the 6‑month follow-up, the girl describes that the support she receives from her mother helped her the best throughout the past 6 months. She feels that her mother respects her sorrows and manages to distract her or calm her down when she feels overwhelmed from grief.

### Triplet B

Triplet B is a rather pragmatic and short-spoken boy who appeared to be reflexive during the assessment.

He never showed elevated levels in scores and subscales of YSR/11-18R (Figs. 1 and 2) and DIKJ. Nevertheless, a slight decrease of reported behavioral problems and depressive symptoms became evident at V2.

Investigation of anxiety (PHOKI) initially revealed an elevated level of fear in the dimension “Fear of Menace” (Stanine = 8), further a borderline value at the total score and at the dimension “Danger and Death” (both Stanine = 7). At V2 anxiety had decreased to Stanine = 4. The value of the dimension “Fear of Medical Intervention” however rose to a marginally elevated level (Stanine = 7). There were no signs of trauma-related symptoms (UPID).

From the mother’s perspective (CBCL/6-18R) triplet B did not exhibit any problems (Fig. 3).

When asked about his grown-up attachment figures triplet B refers to his mother at both times of assessment. He describes a stable, loving and caring relationship, feeling pampered and well looked after. He reports being taken seriously and perceives his mother as being truly interested in his sorrows. He enjoys talking to her and spending quality time with her. He also refers to his grandfather who lives abroad.

### Triplet C

Triplet C was perceived as talkative, emotionally intelligent and thoughtful.

Just like his brother, triplet C did not describe pronounced behavioral problems at either appointment (Figs. 1 and 2). Comparing the outcomes of both dates, a slight decrease of the reported behavioral problems becomes apparent. At V2, triplet C reports marginally elevated “Thought Problems”. DIKJ T‑values also slightly decreased over time. Still, both values were beyond clinical relevance. There was, however, an increase in PHOKI total score with a total PHOKI score at indicating clinically relevant suffering at V2. With view to the subscales, triplet C exhibits symptoms of anxiety in almost all dimensions except for “Social Anxiety” and “Fear of Medical Intervention”. On the scale “Danger and Death”, he even reports to be significantly burdened (Stanine = 9). Triplet C also exhibits symptoms of PTSD at a subclinical range with an elevated UCLA PTSD Reaction Index total score of 16.

The mother describes an overall unproblematic behavior (CBCL/6-18R) (Fig. 3).

When asked about the most important attachment figures triplet C refers to his mother, his grandfather and his siblings. He describes his mother as a loving, stable and caring attachment figure who supported him best throughout the time of grief. He also enjoys having a good time with her. Like his brother, he refers to his grandfather, describes this relationship as stable and enjoys solving problems, exploring nature and learning skilled crafts from his grandfather.

## Discussion

This report about the mourning process of trizygotic triplets (2 boys and 1 girl) shows how anticipated parental loss can in differing grief reactions and mental health outcomes despite objectively equal environmental risk and protective factors. The children investigated shared the same pregnancy, parental home, child rearing conditions and the same event of loss. All three receive the same psychotherapeutic treatment at the same time and describe the mother in a very similar way as a supportive caregiver throughout the bereavement process. The only clear difference between the siblings, besides gender, is the fact that the girl (triplet A) has an intellectual disability and a developmental delay.

Regarding their intrapersonal characteristics and mental health outcomes, these three children could hardly be more different.

Triplet A displays considerably more psychological stress and negative mental health outcomes than her brothers. Triplet B also exhibits different levels of suffering from fear and triplet C shows subclinical trauma-related symptoms and symptoms of anxiety that are, contrary to his siblings, becoming worse over time. Still, the two boys clearly cope in a more functional way.

Apparently contributing to the trauma-associated symptoms of triplet A were reported compulsive and recurrent intrusive thoughts about her father’s buried body. According to her account, she had not perceived any terrifying pictures throughout the father’s illness or the process of dying. Nevertheless, in her case, the sole imagination of her father’s buried body appears to be traumatizing. Given her developmental delay, it can be assumed that her reasoning is still more concretistic and she has a more trivial understanding of death than other children the same age. Hence, learning about her father’s death and about the ritual of the burial lead to the clinically relevant symptoms of distress.

Deduced from these results, it seems most likely that the intellectual disability diagnosed in triplet A is the most pronounced risk factor to develop pathologic grief- and trauma-related psychopathology.

Even though there is still a demand on research on pathological grief reactions in typically developing children, efforts were made to examine their understanding of death and their reactions to the loss of a loved one. Children with developmental disabilities or ID, however, still seem to be neglected in this field of research and there are no instruments for the appropriate assessment or evaluation of grief-related symptoms [14]. This is especially crucial as children and adolescents with ID appear to be more vulnerable to mental health problems [15]. Even in adults the inclusion of bereaved people with ID in mourning rituals or their education about the death of a loved one and its circumstances is poor [14, 16–18] and this extensive lack of knowledge about grief and related symptoms in subjects with ID as well their demand on support renders this population disenfranchised mourners [16].

Clinicians and therapists working with minors with ID in the context of loss should consider developmental, cognitive and emotional aspects. Furthermore, professionals ought to consider the limited ability of abstract thinking in children with ID and their need for explicit and understandable education about death, its circumstances and subsequent rituals [18, 19].

## Conclusion

This report clearly emphasizes the individual nature of grief and resilience. It shows that developmental disability and associated difficulties regarding the understanding of the event or the impaired capacity to cope seems to be a major risk factor in the adaptation to the loss of a loved one. An impaired ability to understand death as well as missing emotional skills to express distress may lead to complicated grieving processes in children and adolescents [14]. Still, knowledge on mourning and bereavement-related symptoms or factors facilitating resilience in individuals with developmental disability is scarce. Further research is needed to promote understanding of grieving processes in this population, with the objective of generating evidence-based guidelines concerning grief education and psychological care for subjects with developmental disability.

## Supplementary Information

Supplement 1 Instruments
