# Supplementary material for: The nature and nurture of resilience—reactions of trizygotic triplet minors to their father’s death
Source: Neuropsychiatr. 2022 Oct 27;37(3):156–61. doi: 10.1007/s40211-022-00434-z (PMC10491695; doi:10.1007/s40211-022-00434-z)
Supplement: Supplementary file 1 — Supplement 1 Instruments [file 40211_2022_434_MOESM1_ESM.docx]

**Instruments**

*CBCL/6-18R and YSR/11-18R*

The Youth Self Report (YSR/11-18R; Döpfner et al., 2014) is a self-rating questionnaire while the Child Behavior Checklist (CBCL/6-18R; Döpfner et al., 2014), is completed by an adult attachment figure. Both questionnaires are comprised of two parts: competence-scales and problem-scales, both evaluating behavior over the last six months. Results are summarized in the subscales and the subscales “Anxious and Depressed”, “Withdrawn and Depressed” and “Somatic Problems” are factored into the superordinate broadband score “Internalizing Problems” while the broadband score “Externalizing Problems” combines the scores of the subscales “Aggressive Behavior” and “Rule Breaking Behavior”. Both broadband scores and the remaining narrowband scales (“Social Problems”, “Thought Problems”, “Attention Problems”) are introduced into the Total Problem Score.

*UPID*

The UCLA PTSD Reaction Index (UPID) is assessing the experience of potentially traumatic events and psycho-reactive symptoms. The questionnaire comprises three parts. The first part provides a list of potentially traumatic events. If a child refers to one or more potentially traumatic experiences, the second part investigates the subjective experience of a potential traumatic event. The third part analyzes the experience and the intensity of listed trauma related symptoms. The total score provides evidence of the presence of PTSD in a view to the DSM-IV and ICD-10 criteria (Steinberg et al., 2004).

*DIKJ*

The DIKJ (Depression inventory for Children and Adolescents) investigates symptoms of depression in children and adolescents aged 8 to 16 years. The evaluation of all items provides one overall T-value describing clinical relevance related to the number and intensity of reported depressive symptoms. A T-value between 60 and 69 reveals marginal suffering, T-values above 70 indicate that the level of suffering from depressive symptoms is clinically relevant. (Stiensmeier-Pelster et al., 2000).

*PHOKI*

PHOKI (Phobia inventory for Children and Adolescents) assesses different dimensions of fear common in children and adolescents aged 8 to 18 years using seven sub-scores delineating different domains of fear that are summarized to a total score. The results are reported as Standard-Nine-values (“Stanine”). Stanine-values from 8 to 9 indicate clinically relevant suffering (Döpfner et al., 2006).

**References:**

1.Döpfner, M., Plück, J. & Kinnen, C. (2014). CBCL/6-18R, TRF/6-18R, YSR/11-18R. Deutsche Schulalter-Formen der Child Behavior Checklist von Thomas M. Achenbach. Göttingen: Hogre-fe.

2.Steinberg, A. M., Brymer, M., Decker, K., Pynoos, R. S. (2004). The University of California at Los Angeles Post-Traumatic Stress Disorder Reaction Index. Current Psychiatry Reports, 6: 96-100

3.Stiensmeier-Pelster, J., Schürmann, M. & Duda, K. (2000). Depressions-Inventar für Kinder und Jugendliche (DIKJ). Handanweisung (2., überarb. u. neunorm. Aufl.). Göttingen, Wien: Hogrefe.

4.Döpfner, M., Schnabel, M., Goletz, H., Ollendick, T.H. (2006). Phobiefragebogen für Kinder und Jugendliche (PHOKI) (1. Aufl.). Göttingen, Wien [u.a.]: Hogref
